# Potential to Eradicate Cancer Stemness by Targeting Cell Surface GRP78

**DOI:** 10.3390/biom12070941

**Published:** 2022-07-05

**Authors:** Hsin-Ying Chen, Ann-Joy Cheng

**Affiliations:** 1 Department of Gastroenterology and Hepatology, Chang Gung Memorial Hospital, Kweishan, Taoyuan 33305, Taiwan; chen.hsinying42@gmail.com; 2 Department of Medical Biotechnology and Laboratory Science, College of Medicine, Chang Gung University, Taoyuan 33302, Taiwan; 3 Graduate Institute of Biomedical Sciences, College of Medicine, Chang Gung University, Taoyuan 33302, Taiwan; 4 Department of Radiation Oncology, Chang Gung Memorial Hospital, Linkou, Taoyuan 33305, Taiwan

**Keywords:** GRP78, cancer stem cells, cancer stemness, cell cycle, cell division

## Abstract

Cancer stemness is proposed to be the main cause of metastasis and tumor relapse after conventional therapy due to the main properties of cancer stem cells. These include unlimited self-renewal, the low percentage in a cell population, asymmetric/symmetric cell division, and the hypothetical different nature for absorbing external substances. As the mechanism of how cancer stemness is maintained remains unknown, further investigation into the basic features of cancer stemness is required. Many articles demonstrated that glucose-regulated protein 78 (GRP78) plays a key role in cancer stemness, suggesting that this molecule is feasible for targeting cancer stem cells. This review summarizes the history of finding cancer stem cells, as well as the functions of GRP78 in cancer stemness, for discussing the possibility of targeting GRP78 to eradicate cancer stemness.

## 1. Introduction

Cancer has been a high-ranked cause of death worldwide for many years [1], and there are no therapeutic strategies that effectively prevent metastasis and tumor relapse in all cancer types. With the disappointing outcomes of conventional therapeutic strategies [2], the novel explanations for the occurrence and progression of tumors are constantly being investigated for developing precise methods to eradicate tumors. Ever since the hypothesis of cancer stemness was proposed, the mechanisms of tumorigenesis and carcinogenesis have begun to lose their secret veil. Multiple cancer models demonstrated the existence of cancer stem cells [3,4,5,6,7], and the objective of eradicating tumors is believed to be achievable in the future. Due to the diverse background, concepts, and focus of different research groups, tumorigenic cells are called cancer stem cells or tumor-initiating cells, and different methods also have been utilized to investigate the various aspects of cancer stemness. This review summarizes the early history of the research of normal and cancer stemness, and the current status of GRP78 studies, serving the purpose of discussing the roles of GRP78 in stemness and the possibility of cancer eradication via targeting GRP78.

## 2. The History of Stem Cell Investigation and Evidence of the Existence of Cancer Stem Cells

Following the term “stem cell” created for describing the originator unicellular organism from which multicellular organisms evolved [8,9], the scientists in the fields of embryogenesis and hematopoiesis took the lead using this term to name the high-hierarchical undifferentiated cells in their research models [9,10,11,12]. In 1896, Artur Pappenheim also applied this term and proposed a common precursor for red and white blood cells [13]. However, the existence of the hematopoietic stem cell could not be experimentally demonstrated and reported until 1961 [14]. Nowadays, stem cells are the ideal model for multiple diseases and cell therapy research. To investigate pathological mechanisms and therapeutic strategies, the research scope of stem cells has been expanding.

The achievement of current stem cell research has three milestones. The first milestone was the earliest model of stem cells beginning with a reprogramming type of experimental design using somatic nuclear transplantation. The first nuclear transplantation model using Northern Leopard Frog was reported in the 1950s, and more than 50% of the transplanted nuclei from advanced blastulas or early gastrulas into recipient enucleated frog eggs developed into frog embryos [15]. This successful demonstration led to the Nobel-Prize-awarded work, in which the successful embryo development rates of nuclear transplantation from early frog embryos were compared to that from differentiated intestine epithelial cells of tadpoles [16,17]. Dolly the sheep and Carbon Copy the cat were the cloned mammals resulting from nuclear transplantation from adult somatic cells [18,19]. Hereafter, these animal cloning models opened the door for the research of embryonic stem cells and inducible pluripotent stem cells.

The second milestone of stem cell research is the investigation of embryonic stem cells originating from the need to understand immune diseases, transplantation, tumorigenesis, and carcinogenesis, as well as the ineffective outcome of therapeutic strategies for cancers. To avoid the ethical issues of using human embryos and the welfare consideration of using animals, teratomas and teratocarcinomas are an applicable resource to establish cell lines for embryonic stemness study models. Steven et al. took the lead and generated an in vivo model of embryonic stemness by generating a transgenic mouse strain prone to spontaneous testicular teratomas [20]. For an authentic and continuous resource of embryonic stem cells, Dr. Brinster spent more than 10 years establishing in vitro methods of cultivating mouse embryos and produced the first mouse chimera bearing a different fur color that was derived from injected blastocyst cells [21,22,23,24]. Evans et al. further generated the first pluripotent stem cell line (which actually developed into tumors after being transplanted into mice) from the culture of mouse blastocysts [25], providing an efficient and easy model for studying the molecular mechanisms of embryonic stemness. For example, Niwa et al. were the first to report that Leukemia Inhibitory Factor (LIF) is required to maintain pluripotency of mouse embryonic cells using a mouse embryonic stem cell line [26]. However, the applications of embryonic stemness research inevitably face the challenge of politics and ethics [27,28,29,30,31,32,33,34,35], evincing the need for experimentally manipulatable pluripotency models.

The third milestone of stem cell research is the establishment of inducible pluripotent stem cells (iPSCs) by Takahashi et al. relying on a collection of research outcomes investigating the many transcription factors of embryonic stem cells. Without the aim of high throughput results of omics platforms, Takahashi et al. defined that “Yamanaka 4 factors”, including Oct3/4, Sox2, c-Myc, and Klf4, were the essential transcription factors required for reprogramming differentiated mouse fibroblasts, from both adult and embryonic, into pluripotent stem cells [36]. Thereafter, Yamanaka 4 factors became the paradigm of in vitro and in vivo pluripotency studies, and these four molecules have also been studied in the field of cancer stemness [7,37,38,39,40,41]. Besides iPSC, stimulus-triggered acquisition of pluripotency (STAP) was another approach that attempted to experimentally manipulate pluripotency. Obokata et al. withdrew their published articles as their results were not reproducible and an investigation was undertaken by the Research Integrity Board of Institute of Physical and Chemical Research (Riken). Their research stated that a short incubation of weak acid followed by treatment with LIF was able to induce pluripotency in mouse somatic cells (references not cited). However, acidic extracellular pH was shown by another research group to influence cell fate determination of a model of acute T cell leukemia [42].

Cancer stemness and normal stem cells have an intertwined history of identification [43,44,45]. The original concept of cancer stem cells was first initiated and described in a pathology textbook authored by a pathologist, Rudolph Virchow, in 1855. Using a regular light microscope, Virchow noticed the histological similarity between a developing fetus and teratocarcinoma and suggested that tumors arose from embryo-like cells [46]. Francesco Durante also used a light microscope to observe cancer and embryonic organ cells which were from the same tissue of origin, and he briefly recorded that his finding was similar to Virchow’s [47]. In a later version of that pathology textbook, Julius Friedrich Cohnheim elaborated on Virchow’s original concept as the “Embryonal-Rest Hypothesis”, speculating that cancer results from the activation of dormant embryonic-tissue remnants in an individual [48]. This view was not well studied until James Till and Ernest McCulloch postulated the existence of hematopoietic stem cells after transplanting bone marrow cells into the mice after lethal irradiation exposure, which is the first evidence of somatic stem cells in grown individuals [14]. Bruce et al. reported the first direct evidence of cancer stem cells using serial dilution, which composed 1–4% of the total population of a mouse lymphoma cell line, were able to propagate the spleens of irradiated recipient mice, showing a similarity or a possible causative relationship between normal and cancer stem cells [6]. Finally, when the cell sorting technique and related antibodies became available in the 1990s, Bonnet et al. demonstrated that CD34^++^CD38^−^ acute myeloid leukemia cells exhibit the main feature of cancer stem cells [5].

**Figure 1:**
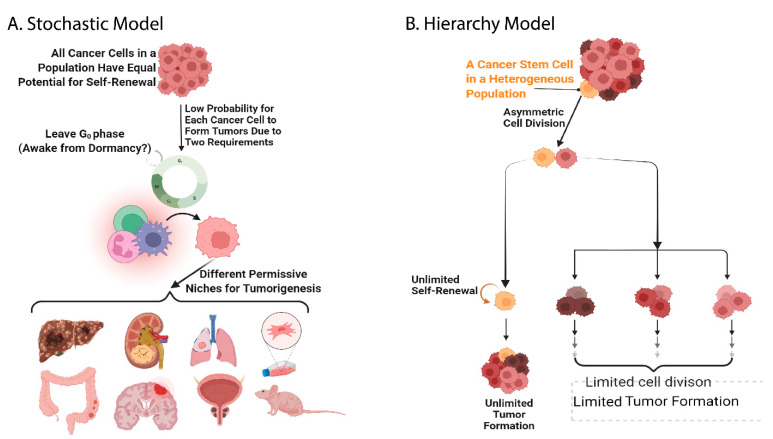
The Hierarchy model and the Stochastic model are the two main hypotheses addressing cancer stemness. Owing to a lack of research investigations referring to the Stochastic model (**A**), this hypothesis is facing the challenge of being combined with the Hierarchy model (**B**) [50]. However, another hypothesis overriding these two relies on the novel ideas or state-of-art experimental designs or platforms that can evaluate the Stochastic model, leading to the in-depth understanding of how these two current models explain all aspects of cancer stemness.

Over recent decades, there have been rapidly accumulating articles supporting the hypothetical existence of cancer stem cells, and two main hypotheses have been proposed to postulate the identity of cancer stem cells. The first hypothesis, the Hierarchy model, postulates that the cancer cells in a population are heterogeneous and only a low percentage of the population, which are called cancer stem cells or tumor-initiating cells, have the capacity for self-renewal to maintain pluri- or multi-potency-like capability, which means the capacity for unlimited cell division and proliferation without the fate of differentiation or senescence and death. Self-renewal to maintain cancer stemness is hypothetically achievable via asymmetric cell division, during which two offspring cells possessing different cell fates are generated. One of the offspring cells is an identical copy to the mother cell (maintaining the stemness), the other does not inherit the cancer stemness but does possess multipotency to differentiate into multilineage progenies. The progenitors that do not inherit cancer stemness are hypothetically not capable of performing asymmetric cell division and therefore only undergo symmetric cell division resulting in identical daughter cells that do not possess cancer stemness either. The other hypothesis, the Stochastic model, claims that all cancer cells in a population have an equal ability to undergo self-renewal, but the probability of each tumor cell entering a cell cycle and finding an optimal niche for performing self-renewal and tumor growth is low (Figure 1) [43,49]. An attempt to challenge the boundary between the Hierarchy and Stochastic model by merging these two hypotheses was proposed [50]. However, the understanding of the essential differences between these two hypotheses will provide solid reasoning and evidence to establish a novel hypothesis overriding these two. This will likely be after convincing data referring the Stochastic model can be demonstrated.

Perhaps due to the currently available investigation strategies and platforms, more articles supporting the Hierarchy model [3,6,7,51,52,53] than the Stochastic model [54,55,56] are published. Most articles use the mathematical method (serial dilution) and cell sorting for experimental evaluation in the Hierarchy model [3,6,7], but a few research groups have reported their findings using hypothetical features of cancer stemness, including cell division pattern and cell cycle phase distribution, in the scope of the Hierarchy model [7,57]. Hopefully, these other features of cancer stemness can be examined in more cancer models in the future. Meanwhile, research designs should be encouraged to develop evaluations for determining whether the Stochastic hypothesis can be discerned in any processes or characteristics of tumorigenesis and carcinogenesis. Once more scientific methods and equipment are developed, further in-depth information and evidence will be reported to decipher the mechanism of cancer stemness maintenance.

## 3. Identification of Cancer Stem Cell Surface Markers in Different Cancer Models

The identification of cell surface markers has been the goal of many cancer stemness research projects, and it seems that currently there is no satisfying answer for all cancer types. As it was the first cancer model to demonstrate the existence of cancer stem cells, leukemia has the longest history of investigation for targeted therapy and cancer stemness [6]. Since CD44 and CD24 are expressed on the cell surface of the lymphocytes homing to the thymus during hematopoiesis [58,59], these two molecules were considered possible markers for diagnosis or target therapy. However, an article in 2003 demonstrated that CD44 and CD24 are viable cell surface markers for identifying breast cancer stem cells by in vivo xenograft formation of serially diluted xenograft tumor cells derived from multiple passaging in mice [3]. This finding led to a long trend of many cancer research projects to evaluate whether CD44 and CD24 can be cancer stem cell markers in other solid cancer models using several approaches. For example, CD44^+^CD24^−^ prostate cancer cells exhibited anchorage-independent in vitro self-renewal and formed xenografts in NOD/SCID mice when merely 100 cells were implanted [60]. CD44^+^CD24^−^ ovarian cancer cells showed enhanced invasion, differentiation, and chemo-resistance [4]. CD44^+^CD24^−/lo^ cell populations of multiple breast cancer cell lines demonstrated enhanced migration/invasion ability and a high correlation to asymmetric segregation of template DNA strands [61], which is one of the proposed mechanisms to achieve self-renewal of cancer stemness. CD44^+^CD24^−^GRP78^+^ head and neck cancer cells exhibit strong capabilities of tumorigenesis, chemo-radioresistance, and invasion [62], fitting the hypothesis that cancer stem cells possess the properties of unlimited self-renewal and enhanced motility across different niches. Although CD44 and CD24 were not concluded as the optimal cancer stem cell markers for lung adenocarcinoma cells, CD44^+^CD24^−/lo^ A549 cells also showed mildly enhanced anchorage-independent in vitro self-renewal [63]. CD44^+^CD24^−/lo^ was one of the heterogeneity phenotypes exhibited by two of the four single cell-derived subclones from a glioblastoma patient [64].

Other more common cell surface markers for cancer stem cells include, among others, CD133, SSEA family members [65], and GRP78 [7,51,62]. CD133 is a membrane glycoprotein expressed in a variety of normal stem cells. This penta-spanned transmembrane glycoprotein interacts with the Wnt/β-catenin and PI3K-Akt signaling pathways and is involved in multiple tumor functions, including metastasis, metabolism, tumorigenesis, drug resistance, and apoptosis [66]. While injecting 10^5^ CD133^−^, cells failed to form any tumors, intracranial implantation of merely 100 CD133^+^ cells isolated from tissues of medulloblastoma and glioblastoma multiforme in human patients formed tumors in NOD-SCID mouse brains [67]. Compared to the CD133^−^ cells, CD133^+^ cells from tissue samples of non-small cell lung cancer patients and lung cancer cell lines had higher expressions of Oct-4 and better in vitro and in vivo self-renewal capability [68]. Stage-specific embryonic antigens (SSEAs) are a family of glycosphingolipids expressed as a part of the cell membrane of embryonic stem cells. SSEA3 and SSEA1 (CD15) were detected in CD45^−^CD44^+^CD24^−^ breast cancer stem cells [69], and brain tumor stem cells [70], respectively. Cell surface GRP78^+^ head and neck cancer cells also displayed better in vivo self-renewal capability than csGRP78^−^ cells [51]. It is important to investigate the different roles of these cell surface markers between normal stem cells and cancer stemness since these cell surface markers are also expressed on normal stem cells [65].

## 4. The Conventional Functions of GRP78, and Following Its Emerging Role in Cancer Stemness

GRP78 has an interesting history regarding its finding and naming. This molecule was originally observed and described in 1974 by Stone et al. For the purpose of generating a vaccine against the avian RNA tumor viruses, they described a plasma membrane protein of about 73 kDa that was upregulated in chick embryo fibroblasts transformed with Avian Sarcoma Viruses [71]. Until 1976, scientists had considered this 73 kDa membrane protein as a specific molecule related to virus transformation/infection. However, in 1977, Shiu et al. demonstrated that glucose starvation upregulated this 78 kDa membrane protein (analyzed by a better protein marker) in both immortalized and Rous virus-transformed chick embryo fibroblasts. Therefore, they concluded that this membrane protein was not related to viral transformation but was rather related to glucose regulation in cells, and thus it was named Glucose-Regulated Protein 78 (GRP78) [72]. Coincidentally, Haas et al. discovered an immunoglobulin heavy chain binding protein, named BiP, also weighing 78 kDa [73]. After cloning the cDNA of BiP, Haas et al. found that BiP belongs to the 70 kDa heat shock protein family [74] and participated in immunoglobulin chain synthesis and defective protein degradation in the endoplasmic reticulum [75,76]. Finally, Haas et al. clarified that BiP is GRP78 in a review publication [77]. Thereafter, it is known that GRP78 is not specific to glucose depletion nor virus infection. It is a resident chaperone in the endoplasmic reticulum whose physiological function is to facilitate normal protein production. GRP78 overexpression/upregulation is correlated with many stress/pathological conditions, such as hypoxia, radiation/ultraviolet exposure, immune diseases, low pH conditions, and most importantly, tumor malignancies [78,79,80,81].

There are many articles demonstrating a relationship between the expression levels of GRP78 protein and the severity of cancers and other diseases. Being the dominant resident chaperone in the endoplasmic reticulum and the main regulator functioning in the unfolded protein response in multiple physiological or pathological stress conditions, GRP78 has consistently been demonstrated to be upregulated, providing a protective effect and/or an essential function in multiple disease models. Total cellular levels of GRP78 received the most attention and investigation in the cancer models. GRP78 was found to be upregulated in prostate and head and neck cancers [81,82], and higher levels of GRP78 protein correlated with a poor prognosis of patients with these and lung cancers [82,83,84]. Importantly, hyper-expression of GRP78 in patients with head and neck cancers was demonstrated to be correlated with the malignancy levels of several oral diseases, as well as with the malignancy-free survival rates of precancerous oral diseases [84], highly supporting that cell surface GRP78 is a significant molecule for targeting cancer stem cells. GRP78 silencing also compromised the in vitro ability of migration and invasion, as well as in vivo tumor growth of head and neck cancer cells [51,81]. GRP78 downregulation enhanced the sensitivity to chemotherapy drugs of multiple types of cancer cells and tumor endothelial cells, including head and neck, breast, lung, colon, glioma, and bladder cancers [62,85,86,87,88,89,90,91]. Compared to the tissues of patients with benign ovarian tumors, malignant ovarian cancers have upregulated expression of GRP78 mRNA [92]. GRP78 was also studied in diseases other than cancer models. In a cardiac hypertension model, GRP78 protein expression was increased in cardiomyocytes after high blood pressure was induced in mice, and exogenous overexpression of GRP78 in cardiomyocytes enhanced hypertrophic growth of cardiomyocytes via the activation of GATA-Binding Protein 4, intensifying the level of cardiac vessel hypertension [93]. In the research project on type 2 diabetes mellitus in a Chinese population, GRP78 was detected in the circulating blood of these patients and circulating GRP78 also correlated with the severity of this kidney disease [94].

GRP78 expression on the cell surface of human rhabdomyosarcoma cells treated with thapsigargin and GRP78 autoantibody detected in normal human peripheral blood [95] suggested a potential signaling role of this molecule. GRP78 autoantibodies purified from the serum of prostate cancer patients exhibited pro-proliferative effects and increased intracellular calcium levels in cell lines of prostate cancer and melanoma; this autoantibody was shown to specifically recognize a tertiary structural motif mimicking an epitope in GRP78 [96]. Thereafter, cell surface GRP78 was hypothesized to function as an oncogenic signaling receptor, and the ligands of this hypothetic signaling receptor and the signaling outcomes have been extensively explored. The potential molecules associated with or binding to GRP78 were reported by multiple research groups, yet different ligand associations with cell surface GRP78 result in different signaling outcomes. For example, α2-macroglobulin [97] and Cripto [98] were shown to associate with cell surface GRP78 in prostate cancer cells and promoted signaling pathways of proliferation, metastasis, and tumor growth. When Par-4, TRAIL (tumor necrosis factor related apoptosis inducing ligand) [99] and recombinant Kringle 5 of human plasminogen [100] associated with cell surface GRP78 in prostate cancer, endothelial, and glioma cells, apoptosis was induced. GRP78 was reported as an autoantigen for B and T cells in rheumatoid arthritis patients [101,102]. Entry into the host cells of several virus strains, including Coxsackie virus A9 [103], Borna disease virus [104], severe acute respiratory syndrome coronavirus 2 (SARS-CoV-2, which causes COVID-19) [105], depended on GRP78 expressed on the cell surface of the host cells. Although it could be certain that GRP78 plays important roles in various cellular events, it was also questionable why a resident chaperone expressed in the endoplasmic reticulum in most of the somatic cells was reported to function as a signaling receptor in another subcellular compartment.

The molecules associated with cell surface GRP78 were never comprehensively and systematically investigated before Chen et al. reported their findings. Using the newly designed quantitative mass spectrometry platform, they detected and verified multiple endogenous interactome candidates of cell surface GRP78 and intracellular GRP78 in head and neck cancer cells [7]. There are also other articles reporting other molecules associated with cell surface GRP78 or intracellular GRP78 [80,81,82,83,84]. Collectively, these sound demonstrations consolidate that GRP78 still functions as a chaperone in the plasma membrane compartment. If the endogenous interactome of GRP78 in different subcellular compartments of other cancer models can be identified by the quantitative platform of mass spectrometry, not only will it provide the molecular machinery of malignancy phenotypes, but it will also provide detailed molecular profiles of cancer stemness due to GRP78’s roles in cancer stemness discussed below.

**Figure 2:**
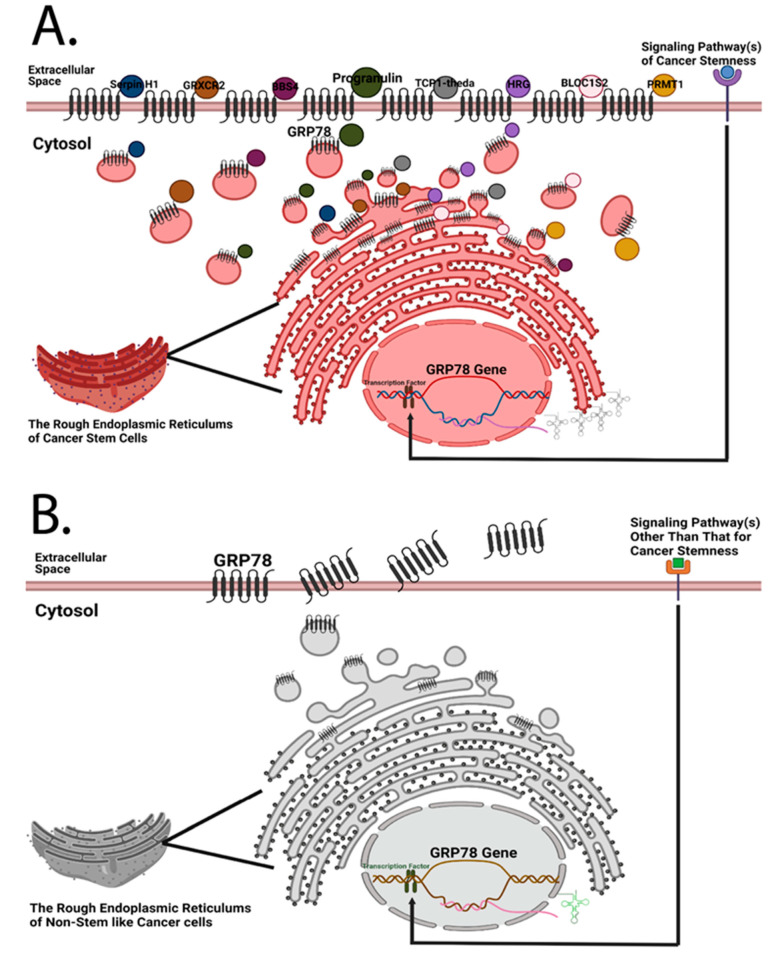
Both cancer stem cells and non-stem-like cancer cells are hypothesized to express cell surface GRP78, but the amounts of cell surface GRP78 and its interactome of these two types of cells are different. (**A**) Previously, Chen et al. designed a quantitative mass spectrometry platform to detect the interactome of cell surface GRP78 in head and neck cancer cells [7]. Given that GRP78 silencing has a regulatory influence on stemness-related markers [7], it is logical to hypothesize that cancer stem cells possess a more diverse GRP78 interactome than non-stem-like cancer cells; and that cancer cells expressing a higher amount of cell surface GRP78 may possess the highest hierarchical rank of cancer stemness. (**B**) Due to the higher proliferation rate of cancer cells, an increase in plasma membrane synthesis in the endoplasmic reticulum is required, resulting in the translocation of resident chaperone GRP78 to the cell surface. Under this circumstance, non-stem-like cancer cells may express a certain amount of cell surface GRP78 even if there are no plasma membrane-bound proteins that need to be chaperoned to the subcellular compartment of the plasma membrane. GRP78 is likely secreted afterwards.

**Figure 3:**
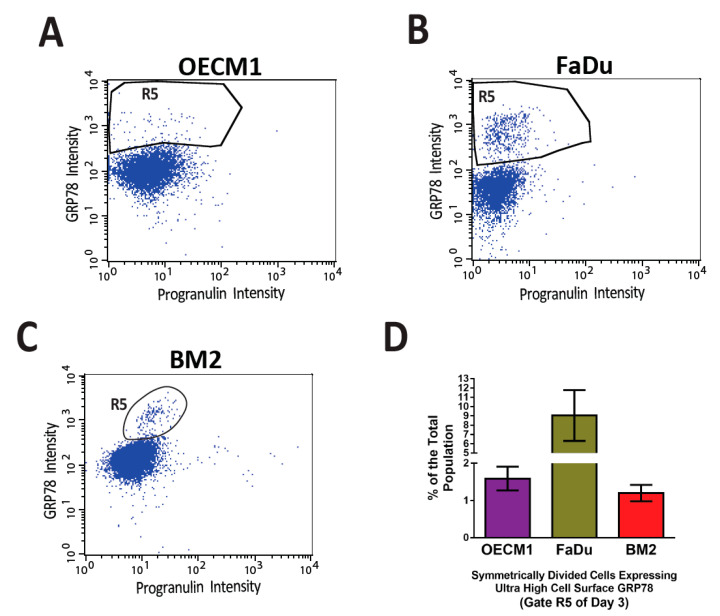
Distinct small populations of symmetrically divided cancer cells expressing ultra-high levels of cell surface GRP78 were consistently observed in the flow cytometry-based cell division assay. In a previously published article by Chen et al. [7], a high throughput flow cytometry-based cell division assay was developed to characterize the profile of asymmetric/symmetric cell divisions in the heterogeneous cell populations of three head and neck cancer cell lines, OECM1 (**A**), FaDu (**B**), and BM2 (**C**). In the three cancer cell lines that went through symmetric cell division (Gate R3 of Figure 5B,D,F of reference [7]), distinct cell populations expressed ultra-high levels of cell surface GRP78 (the R5 gates) consistently above the levels of major csGRP78 positive populations. (**D**) The quantification of the R5 gates of the three head and neck cancer cell lines. The percentages of R5 gates are 1–9% of the total cell populations.

The previous findings of GRP78 led to the suggestion that GRP78 plays a role in cancer stemness. GRP78 overexpression in Chinese Hamster Ovary cells increased their resistance to Etoposide treatment [85]. Since Etoposide hampers the function of DNA polymerase, and cell division pattern and frequency are the characteristics within the scope of cancer stemness, this finding is the first to show that GRP78 can protect somatic stem cells from apoptosis. This is also an indirect suggestion that GRP78 may have functions in cancer stemness because of the possibility of the tumorigenic origin of somatic stem cells. Then, Wu et al. were the first to demonstrate the possibility of cell surface GRP78 serving as a cancer stem cell marker. They showed that the head and neck cancer cell population of cell surface GRP78^hi^ (csGRP78^hi^) had better tumorigenesis capability than that of csGRP78^lo^ in the mouse xenograft model determined by serial dilution of the transplanted cell numbers [51], which is one of the traditional methods to evaluate the occurrence, or the level, of cancer stemness. Since serial dilution of the cell number of sorted head and neck cancer cells based on the cell surface GRP78 level has a direct influence on xenograft tumorigenesis in a mouse model, cell surface GRP78 is hypothesized to serve a direct role in cancer stemness. Therefore, Chen et al. further investigated the influence of the expression levels of csGRP78 on other hypothetical properties of cancer stemness [7], including cell division pattern and cell cycle phase profile since asymmetric cell division is considered as a sign of differentiation in cell fate determination [106], and pluripotency state of embryonic stem cells was shown to be maintained in the cell cycle S and G2 phases [107]. They found that the percentages of csGRP78^hi^ head and neck cancer cells in the G2/M cell cycle phase were more than 9-fold higher than that of csGRP78^lo^ in the G1 cell cycle phase. By using the flow cytometry-based cell division assay developed by Chen et al., head and neck cancer cells expressing Progranulin on their cell surface exclusively perform symmetric cell division, while those expressing cell surface GRP78 perform both symmetric and asymmetric cell division. Meanwhile, GRP78 silencing downregulated multiple stemness-related markers. This investigation demonstrates that GRP78 is a chaperone related to cancer stemness maintenance in head and neck cancer cells (Figure 2), considering that multiple interactome molecules to csGRP78 were detected [7]. Furthermore, Chen et al. noticed that there was a distinct cell population expressing an ultra-high level of csGRP78 (Figure 3) in the symmetrically divided csGRP78^+^ cells in three head and neck cancer cell lines (The data presented in this study are available in Gate R3 of Figure 5B,D,F of reference [7]), but it is unknown whether these distinct cell populations possess cancer stemness of the highest rank of the hierarchy.

Besides the head and neck cancer model, the potential function of GRP78 in cancer stemness is also reported in other cancer models. Exogenous expression of GRP78 in MDA-MB-231 cells increased the percentage of cell population expressing CD44^+^/CD24^−^, and cGRP78^+^ MCF7 cells exhibited better in vitro and in vivo tumorigenesis than the total unsorted and the CD44^+^/CD24^−^ population [108]. GRP78 silencing significantly decreased the in vivo survival of glioma stem cells after ionizing radiation [109]. Being chaperoned by csGRP78 for lysosomal degradation, BACE2 silencing suppressed in vitro and in vivo tumorigenesis capability of glioma stem cells, demonstrating that csGRP78 engages in the regulation of cancer stemness in glioma [110]. Interestingly, using a CRISPR knockout system, GRP78 was shown to prime non-small lung adenocarcinoma cells for cell cycle re-entry after Cisplatin treatment, allowing cancer cells to escape the fate of senescence [111]. How cancer stem cells regulate cell cycle progression and initiate the entry or arrest is proposed to be a key cellular event of cell stemness maintenance [7]. The mechanism of how GRP78 contributes to cancer stemness needs to be investigated for precise targeting of cancer stem cells. Several cell-signaling pathways were suggested to be responsible for the regulation of self-renewal in cancer stem cells [112]. Therefore, verifying whether GRP78 regulates these pathways, or vice versa, will be valuable for understanding the mechanism of cancer stemness maintenance.

## 5. GRP78-Based Therapy for Multiple Cancer Models, and Specificity Concerns of GRP78 Targeting

Recently, GRP78-based target strategies for therapy were demonstrated in several cancer models. Antibodies were the initial strategy for targeting GRP78 [113,114]. A clinical therapeutic trial using a monoclonal antibody recognizing GRP78 to treat patients with advanced melanoma was completed [114]. Coating with SP94 peptide that specifically binds to GRP78, nanoparticles containing doxorubicin significantly reduced the sizes of prostate cancer xenografts in mice exposed to ultrasound real-time imaging [115]. Other nanoparticles coated with VAP (a D-peptide ligand of GRP78) [116] and RI-VAP (a specific ligand of cell surface GRP78) [117] were shown to effectively target glioblastoma and glioma, respectively. Hebbar et al. generated T cells expressing chimeric antigen receptors (CARs) which specifically recognize GRP78 (GRP78-CAR T cells) by expressing a peptidic GRP78 ligand in T cells in vitro differentiated from human peripheral blood mononuclear cells (PBMCs) from healthy donors. Their GRP78-CAR T cells showed effective killing activity against the xenografts of acute myeloid lymphoma in mice yet leaving normal hematopoietic progenitor cells unharmed [118]. Salidroside suppressed the growth of nasopharyngeal cancer xenografts in mice, and the mechanism of this suppression was shown to correlate with the downregulation of GRP78 via Salidroside-induced upregulation of miR-4262 [119].

It is unknown whether GRP78 is expressed on the cell surface of normal stem cells, and as a result, the specificity of GRP78-targeted therapy is the main concern for cancer patients. Several reports mentioned or characterized the role of GRP78 in the field of normal stem cells. GRP78 was found to serve an essential role during the early development of mouse embryos and embryo implantation. GRP78 knockout in the mouse embryos resulted in apoptosis of the inner cell mass, and homozygous knockout of GRP78 was lethal to the mouse embryos [120]. GRP78 protein expression was detected in the luminal and glandular epithelia of the mouse uterus during early pregnancy, and a high level of GRP78 was detected at the implantation site of the embryo [121]. In a cartilage development model, GRP78 protein expression was found in the chondrocytes of the growth plates of late-pregnancy mouse embryos and newborns. Additionally, GRP78 overexpression in the undifferentiated mouse embryo fibroblasts and differentiated chondrogenic cells increased the percentages of cells in the S phase in the total population [122]. Intravenous injection of GRP78 siRNA appeared to compromise the stem cell niche of normal mouse hair follicles Fig 6. B-1 of reference [81] . Yang et al. reported that GRP78 overexpression decreased the apoptosis induced by misfolded androgen receptor in mouse embryonic stem cells, and GRP78 knockdown increased the accumulation of androgen receptor aggregates and caspase 3 activity [123]. This shows that GRP78 is an important regulator of lineage determination of differentiating embryonic stem cells. CsGRP78 was demonstrated to promote reprogramming of human neonatal keratinocytes and iPSCs derived from fibroblasts [108]. Further investigation of cell surface GRP78 in the fields of normal stem cells and cell fate determination is required for evaluating the specificity of targeting csGRP78 in cancer therapeutic strategies.

## 6. Conclusions

After the investigation of GRP78 for nearly half of a century, different methods have been invented for targeting this molecule, including antibodies, nanoparticles coated with specific ligand peptides, and specific CAR-T cells. For prolonging the disease-free survival of cancer patients, it is necessary to precisely target the cancer stem cells for eradicating cancers. Therefore, studying the mechanism of how GRP78 contributes to the maintenance of cancer stemness is important for understanding the characteristics of cancer stem cells. Given that ultra-high levels of csGRP78 may represent high-hierarchical cancer stem cells (Figure 2), targeting csGRP78 may still be a feasible therapeutic intervention for eradicating cancers.

## Data Availability

The data presented in this study are openly available Figure 5 at https://doi.org/10.1038/s41598-017-14604-5, 18 May 2022, reference [7].
